# Randomness-based macroscopic Franson-type nonlocal correlation

**DOI:** 10.1038/s41598-022-07740-0

**Published:** 2022-03-08

**Authors:** Byoung S. Ham

**Affiliations:** School of Electrical Engineering and Computer Science, Gwangju Institute of Science and Technology, 123 Chumdangwagi-ro, Buk-gu, Gwangju, 61005 South Korea

**Keywords:** Quantum mechanics, Quantum optics

## Abstract

Franson-type nonlocal correlation is related to Bell inequality violation tests and has been applied for quantum key distributions based on time bin methods. Using unbalanced Mach–Zehnder interferometers, Franson correlation measurements result in an interference fringe, while local measurements do not. Here, randomness-based macroscopic Franson-type correlation is presented using polarization-based two-mode coherent photons, where the quantum correlation is tested by a Hong-Ou-Mandel scheme. Coherent photons are used to investigate the wave properties of this correlation. Without contradicting the wave-particle duality of quantum mechanics, the proposed method provides fundamental understanding of the quantum nature and opens the door to deterministic quantum information science.

## Introduction

Over the last several decades, the Bell inequality^1,2^ has been a guideline and a testing tool for nonlocal quantum correlation in quantum mechanics^3–10^. Franson-type nonlocal correlation has been studied with respect to the Bell inequality violation using noninterfering Mach–Zehnder interferometers (MZIs)^11–13^, where the Franson-type correlation has been widely adapted for quantum key distributions^14–16^ and quantum random number generation^17^ based on time bin methods. Unlike Bell inequality violations based on measurement projections^1^, the Franson correlation uses a polarization-independent interferometric scheme satisfying the randomness of path choices for photon selections in local measurements. On the other hand, nonlocal coincidence measurements between remotely separated parties result in strong correlations between the path choices due to individually correlated photon pairs. Besides, dynamics of diffusion problems have also been discussed using nonlocal effects^18–21^. Here, we present a coherence model of the Franson-type correlation compatible with a macroscopic (coherence) regime using the wave nature of a photon, which is incompatible with the conventional particle nature of quantum mechanics. This coherence interpretation of the quantum correlation does not contradict quantum mechanics of the wave-particle duality, where both features are mutually exclusive^22^. For this, randomness of the original Franson scheme is accomplished by polarization bases in a balanced MZI. Instead of coincidence detection between two remotely separated parties, a Hong-Ou-Mandel (HOM) type detection scheme is used to understand this coherence-based correlation^23^.

Recently, a coherence version of anticorrelation, the so-called a HOM dip has been proposed^24^ to revisit the quantum nature of two-mode entanglement^23^, where anticorrelation is redefined with a definite phase relation between the interfering paired photons on a beam splitter (BS). According to the coherence interpretation of anticorrelation^24^, a new understanding of HOM dips has been established with a specific phase relation between the signal and idler photons, as experimentally demonstrated in a pair of trapped ions^25^. Considering the randomness of photon choices for HOM experiments, however, the fringe in each output port disappears, while coincidence measurements between the two ports shows strong anticorrelation for the paired photons^23,24^. The fundamental physics of HOM dips has also been studied for independent light sources, in which the HOM-type quantum correlations are induced by a coherence control between independent photons^26,27^. Like the entangled photon pairs resulting in no fringes for local measurements, macroscopic quantum correlation has been suggested recently using phase basis randomness in a double MZI scheme^28^. Thus, the HOM type correlation method is appropriate for investigating correlated photon’s characteristics in terms of coherence. Here, the interference fringe of the Franson correlation is interpreted using polarization bases for both the local randomness and quantum correlation.

## Analysis

**Figure 1:**
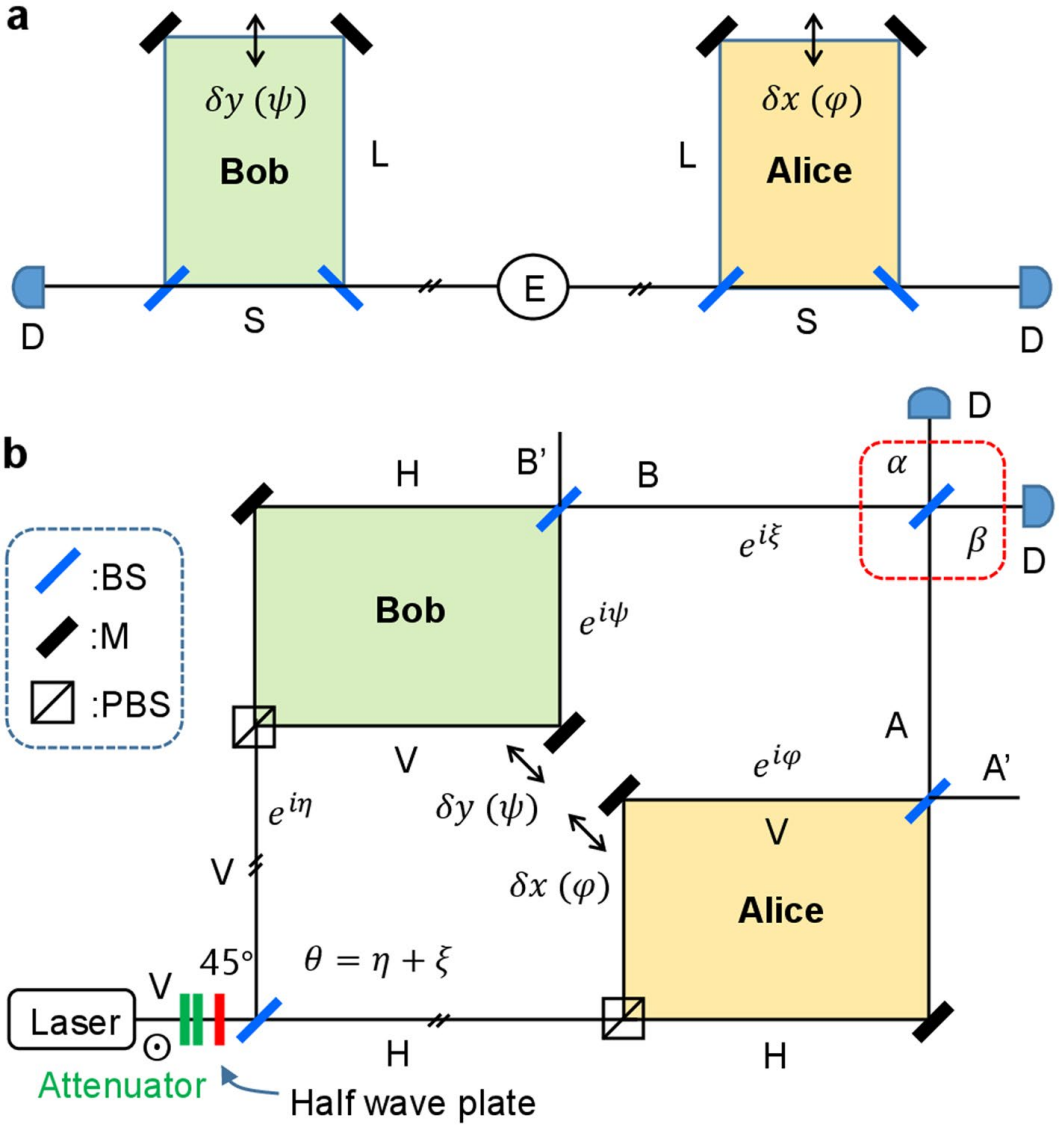
Schematic of Franson-type correlation. (**a**) an original version. (**b**) a coherence version. E: an entangled photon pair generation light source, S/L: short/long optical path, D: detector, BS: nonpolarizing beam splitter, PBS: polarizing beam splitter, H (V): horizontally (vertically) polarized photon. $\mathrm{\varphi }=\frac{2\pi }{\lambda }\delta x$ and $\uppsi =\frac{2\pi }{\lambda }\delta y$, where $\lambda$ is the wavelength of the light. The phases $\upeta$ and $\upxi$ are due to relative path-length difference between Alice and Bob except for the noninterfering PBS-BS MZI in each side. The initial polarization of laser light is vertical (V) with respect to the plane of incidence.

Figure 1a shows the original scheme of Franson-type nonlocal correlation^12,13^, whereas Fig. 1b shows the corresponding coherence version discussed herein. The noninterfering MZIs in Fig. 1a are mimicked by a PBS-BS composed noninterfering MZIs in Fig. 1b, where the short-short (S–S) and long-long (L-L) path superposition in Fig. 1a is replaced by polarization superposition between horizontal (H–H) and vertical (V–V) ones. For proof of principle, a typical interference scheme by a BS (see the red dotted box) is adapted. Here, it should be noted that coincidence measurement in Fig. 1a is a way to trace the nonlocal correlation of coupled photons via quantum superposition between the S–S and L-L paths. For the investigation of the nonlocal correlation, however, the cross-interference scheme of the red-dotted box between correlated photons (A and B) is used to understand coherence features of the Franson correlation. Here, it should be emphasized that Fig. 1b is effective not only for the conventional particle nature of photons, but also for the wave nature of photons due to the coherent control of randomness^28^.

To make it conceptually identical to the original scheme of Franson correlation in Fig. 1a ^12,13^, a 45 degree-rotated half-wave plate (HWP) is used for random polarization bases in both parties with PBS-BS based MZIs as shown in Fig. 1b. Here the 45-degree rotated HWP stands for random polarization photons even if actual rotation is 22.5 degrees. The random generation of polarized photons provides quantum superposition between two independent bipartite orthonormal bases, where coherence between paired bases plays a key role for quantum correlation. Specifically, the present wave nature-based approach can expand the scope of conventional microscopic quantum correlation into a macroscopic one via collective control of individual photons or atoms^29,30^.

Starting from a coherent ensemble of photons generated from the laser in Fig. 1b, neutral density filters (Attenuator) are inserted right after the laser to reconfigure the coherent scheme into a microscopic one composed of doubly-bunched photon pairs governed by Poisson statistics. The ratio of doubly bunched photons to single photons is determined by the mean photon number $\langle n\rangle$ for coherent state $|\alpha \rangle$: $|\alpha \rangle ={e}^{-\frac{{\left|\alpha \right|}^{2}}{2}}\sum \frac{{\alpha }^{n}}{\sqrt{n!}}|n\rangle$. For $\langle n\rangle =0.012$, the double-to-single photon generation ratio has been experimentally demonstrated to be 0.005, where its temporal distribution is nearly sub-Poissonian via coincidence measurements due to the elimination of vacuum and single photon states. Each photon pair is split into two paths for Alice and Bob equally by the first BS, where each photon’s polarization is randomly provided by a half wave plate. This random polarization of H (horizontal) and V (vertical) in Fig. 1b is similar but not identical to the SPDC type-II case, in terms of entanglement^6–10^. Single photons in Fig. 1b do not contribute to the coincidence measurements. Three or more bunched photons are also neglected due to Poisson statistics corresponding to ~ 1% generation ratio to the doubly-bunched photons.

The H-V polarization-based Franson correlation scheme of Fig. 1b is now analyzed using the polarization-basis superposition for the PBS-BS MZIs:

1 $$\left| \Psi \right\rangle_{A} = \left( {\left| {H} \right\rangle_{A} + ie^{i\varphi } \left| {V } \right\rangle_{A} } \right)/\sqrt 2 ,$$

2 $$\left| \Psi \right\rangle_{B} = ie^{i\theta } \left( {\left| H \right\rangle_{B} + ie^{i\psi } \left| V \right\rangle_{B} } \right)/\sqrt 2$$

where the amplitude of H and V is set equivalent to ${E}_{0}$, and the term $i{e}^{i\theta }$ is due to the total relative path-length difference $(\uptheta =\upeta +\upxi )$ between Alice and Bob. For coincidence measurements, the last BS for $\mathrm{\alpha }$ and $\upbeta$ interference (see the red-dotted box) is removed to satisfy the original nonlocal correlation scheme between Alice and Bob in Fig. 1a. The subscript A and B in Eqs. (1) and (2) indicate Alice and Bob, respectively. Due to the Fresnel-Arago law^31^, however, H-V interference terms are automatically removed^32^. Here, the relative phases $\mathrm{\varphi }$ and $\uppsi$ are related with the path-length $(\mathrm{\delta x};\mathrm{ \delta y})$ difference inside the PBS-BS MZIs, which are precisely controlled without violation of the uncertainty principle.

For analysis of the coherence version of Franson correlation in Fig. 1b, we first show single photon-based coincidence measurements and then expand it to a general case of a coherent ensemble. The analytic expression for the coincidence detection ${R}_{AB}$ measured by both detectors remotely located in Alice’s and Bob’s sides is directly obtained from Eqs. (1) and (2):

3 $$\begin{gathered} \left\langle {R_{AB} } \right\rangle = \frac{1}{4}\left\langle {\left( {\left\langle H \right|_{A} - ie^{ - i\varphi } \left\langle V \right|_{A} } \right)\left( {\left\langle H \right|_{B} - ie^{ - i\psi } \left\langle V \right|_{B} } \right)\left( {\left| H \right\rangle_{A} + ie^{i\varphi } \left| V \right\rangle_{A} } \right)\left( {\left| H \right\rangle_{B} + ie^{i\psi } \left| V \right\rangle_{B} } \right)} \right\rangle \hfill \\ = \frac{1}{4}\left\langle {\left( {\left\langle {H|H} \right\rangle_{AB} + e^{ - i(\varphi - \psi )} \left\langle {V|V} \right\rangle_{AB} } \right)\left( {\left\langle {H|H} \right\rangle_{BA} + e^{i(\varphi - \psi )} \left\langle {V|V} \right\rangle_{BA} } \right)} \right\rangle \hfill \\ = \frac{{I_{0}^{2} }}{2}\left\langle {1 + \cos \left( {\varphi - \psi } \right)} \right\rangle , \hfill \\ \end{gathered}$$

where ${I}_{0}={E}_{0}{E}_{0}^{*}$, $\mathrm{\varphi }=\frac{2\pi }{\lambda }\delta x$, and $\uppsi =\frac{2\pi }{\lambda }\delta y$. Thus, Eq. (3) shows the same result as the original Franson-type nonlocal correlation^12,13^. With precise control of $\mathrm{\varphi }$ and $\uppsi$, the coincidence measurement in Eq. (3) shows a definite Bell inequality violation with a visibility greater than $1/\sqrt{2}$^1–16^.

On the other hand, the coherence version of Franson-type correlation in Fig. 1b can be described for the interference of coherent fields A ${(\Psi }_{A})$ and B ${(\Psi }_{B})$ as follows:

4 $$\begin{aligned} I_{\alpha } & = \frac{1}{4}\left\{ {\left[ {\left( {H_{A} + ie^{i\varphi } V_{A} - e^{i\theta } \left( {H_{B} + ie^{i\psi } V_{B} } \right)} \right)} \right]\left[ {\left( {H_{A}^{*} - ie^{ - i\varphi } V_{A}^{*} - e^{ - i\theta } \left( {H_{B}^{*} - ie^{ - i\psi } V_{B}^{*} } \right)} \right)} \right]} \right\} \\ & = \frac{1}{4}\left[ {H_{A} H_{A}^{*} + V_{A} V_{A}^{*} + H_{B} H_{B}^{*} + V_{B} V_{B}^{*} - \left( {H_{A} H_{B}^{*} + V_{A} V_{B}^{*} e^{i(\varphi - \psi )} } \right)e^{ - i\theta } - \left( {H_{B} H_{A}^{*} + V_{B} V_{A}^{*} e^{ - i(\varphi - \psi )} } \right)e^{i\theta } } \right] \\ & = \frac{{I_{0} }}{2}\left[ {2 - \cos \left( \theta \right) - {\text{cos}}\left( {\varphi - \psi - \theta } \right)} \right], \\ \end{aligned}$$

where ${V}_{i}$ and ${H}_{i}$ indicate polarized coherent fields in the Alice’s and Bob’s sides, whose amplitude is ${E}_{0}$: ${\Psi }_{j}=\frac{1}{\sqrt{2}}\left({H}_{j}+{ie}^{i{\zeta }_{j}}{V}_{j}\right); j=H, V; {\zeta }_{1}=\varphi ; {\zeta }_{2}=\psi$. Obviously, the orthogonal polarization bases in Fig. 1 should include circular polarization bases without the 45 $^\circ$ half-wave plate. In this case, a $\uppi$ phase shift in Vs between Alice and Bob can be compensated by the control of $\uppsi$ or $\mathrm{\varphi }$. According to the wave-particle duality, the phase of a photon cannot be compatible with a photon number or an energy state. In other words, the coincidence detection must be replaced by coherence detection. All ${V}_{i}{H}_{j}$ interference terms are deleted due to the Fresnel-Arago law^31^. Each output amplitude superposed by Eqs. (1) and (2) is as follows: $E_{\alpha } = \frac{1}{\sqrt 2 }\left( {\Psi_{A} + ie^{i\xi } \Psi_{B} } \right)$; $E_{\beta } = \frac{i}{\sqrt 2 }\left( {\Psi_{A} - ie^{i\xi } \Psi_{B} } \right)$; $I_{j}$ is defined as ${{I}_{j}=E}_{j}{E}_{j}^{*}$. In the view point of coherence optics, Fig. 1b satisfies a general MZI scheme (see the big square including both noninterfering PBS-BS MZIs). For coherence optics of MZI, $E_{0}$ can be either a single photon^33^ or coherent light governed by Maxwell’s equation. Likewise,

5 $$I_{\beta } = \frac{{I_{0} }}{2}\left[ {2 + \cos \left( \theta \right) + {\text{cos}}\left( {\varphi - \psi - \theta } \right)} \right].$$

**Figure 2:**
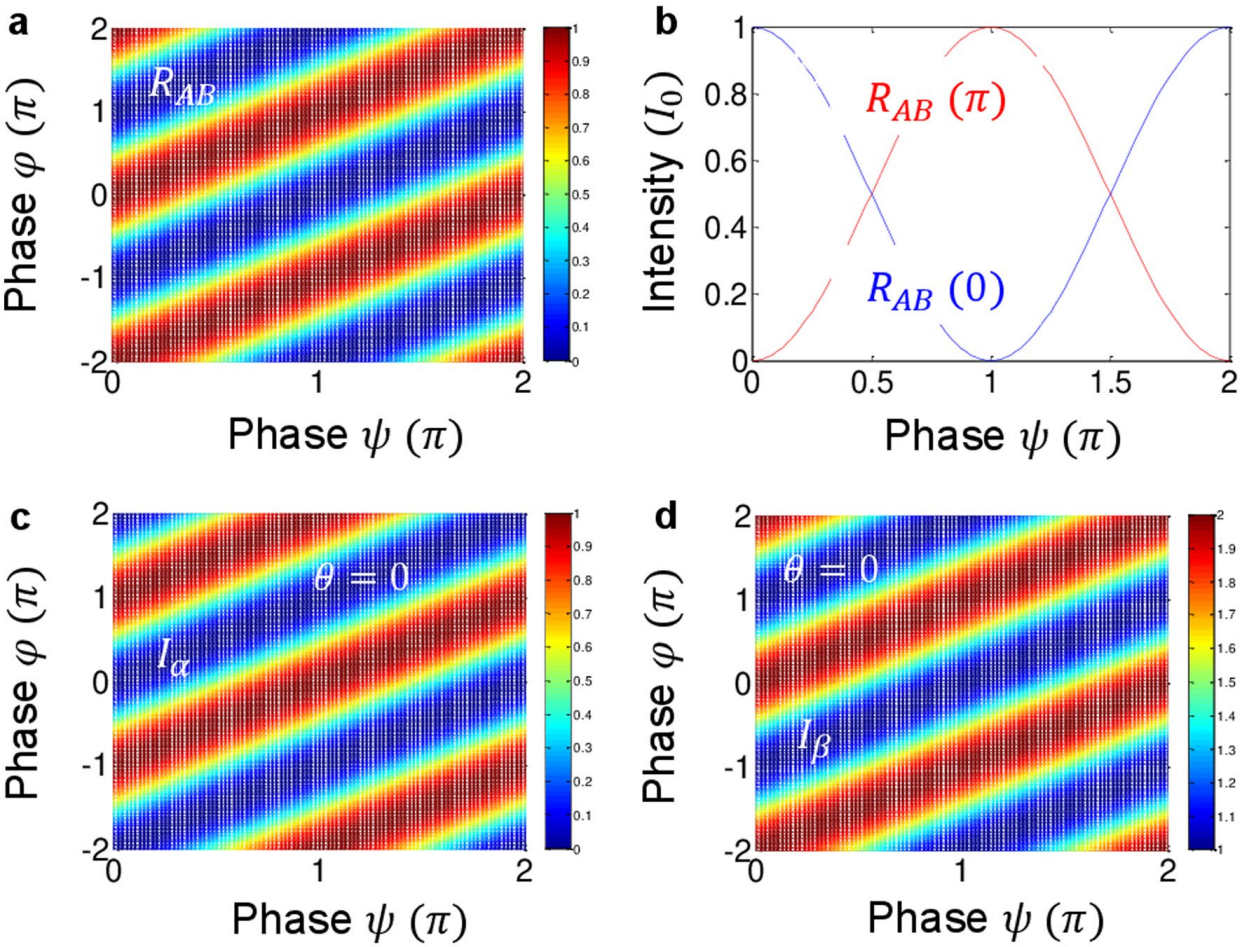
Numerical simulations for Eqs. (3)–(5). (**a**) and (**b**) Normalized ${R}_{AB}$ for $\uptheta =0$. The values in parenthesis are for $\mathrm{\varphi }$. (**c**) and (d) Intensities ${I}_{\alpha }$ and ${I}_{\beta }$ for $\uptheta =0$.

Although Eqs. (4) and (5) are different from Eq. (3), they share the phase sensitive fringe (see Fig. 2)^6,12,32^. Unlike coincidence measurements for single photons, the coherence version of Franson-type nonlocal correlation in Eqs. (4) and (5) includes both classical and quantum limits depending on the relative phase between $\mathrm{\varphi }$ and $\uppsi$ as well as $\uptheta$. In other words, the quantum feature of the coherence version of Franson correlation relies on the relative phase information between the coherent photon pair. In practical situations, however, this phase control between two parties should be conducted in a trusted-device scenario. Such a scenario can be easily obtained by a laser locking-based feedback control^34^. The $\uptheta$ effect resulting from the BS-BS MZI (see the big square in Fig. 1b) is viewed for a HOM dip^23^. Thus, Franson correlation in Fig. 1b has a chance to be compared with an interference model of a HOM dip for the fundamental physics of quantumness or nonclassicality.

Figure 2 shows numerical simulations for Eqs. (3)–(5), where a single-shot near-perfect measurement is another impotant benefit of the coherence (macroscopic) version. Figure 2a,b are single-photon-level coincidence measurements based on Eq. (3) as a reference, whereas Fig. 2c,d are for the coherence counterparts based on Eqs. (4) and (5). In terms of coincidence measurements for the particle nature of photons, the relative phase-dependent ${R}_{AB}$ in Fig. 2b shows a definite coherence feature as observed in ref. 12. If there is no superposition between polarization bases in the noninterfering PBS-BS MZIs, then the interference term in Eq. (3) is negated, resulting in ${\uptau }$ dependent intensity correlation:

6 $$R_{AB} \left( \tau \right) = \left\langle {\left( { - ie^{ - i\varphi } \left\langle V \right|_{A} } \right)\left( { - ie^{ - i\psi } \left\langle V \right|_{B} } \right)\left( {ie^{i\varphi } \left| V \right\rangle_{A} } \right)\left( {ie^{i\psi } \left| V \right\rangle_{B} } \right)} \right\rangle = I_{0}^{2} .$$

Thus, the observed fringe in the original Franson experiment^12^ is rooted in the superposition between S–S and L-L paths of a photon pair. In other words, the quantum feature of Franson correlation is due to the coherence between paired photons via random basis superposition (discussed below).

The output intensities of ${I}_{\alpha }$ and ${I}_{\beta }$ in Fig. 1b are opposite each other, satisfying general MZI physics as shown in Fig. 2c,d. The maxima of ${I}_{\alpha }$ occur at $\uppsi +\uptheta =\mathrm{\varphi }\pm\uppi$ to satisfy MZI physics, whereas ${I}_{\beta }$ at $\uppsi +\uptheta =\mathrm{\varphi }$. For $\uptheta =0$, Fig. 2c,d are identical, as Fig. 2a,b are.

**Figure 3:**
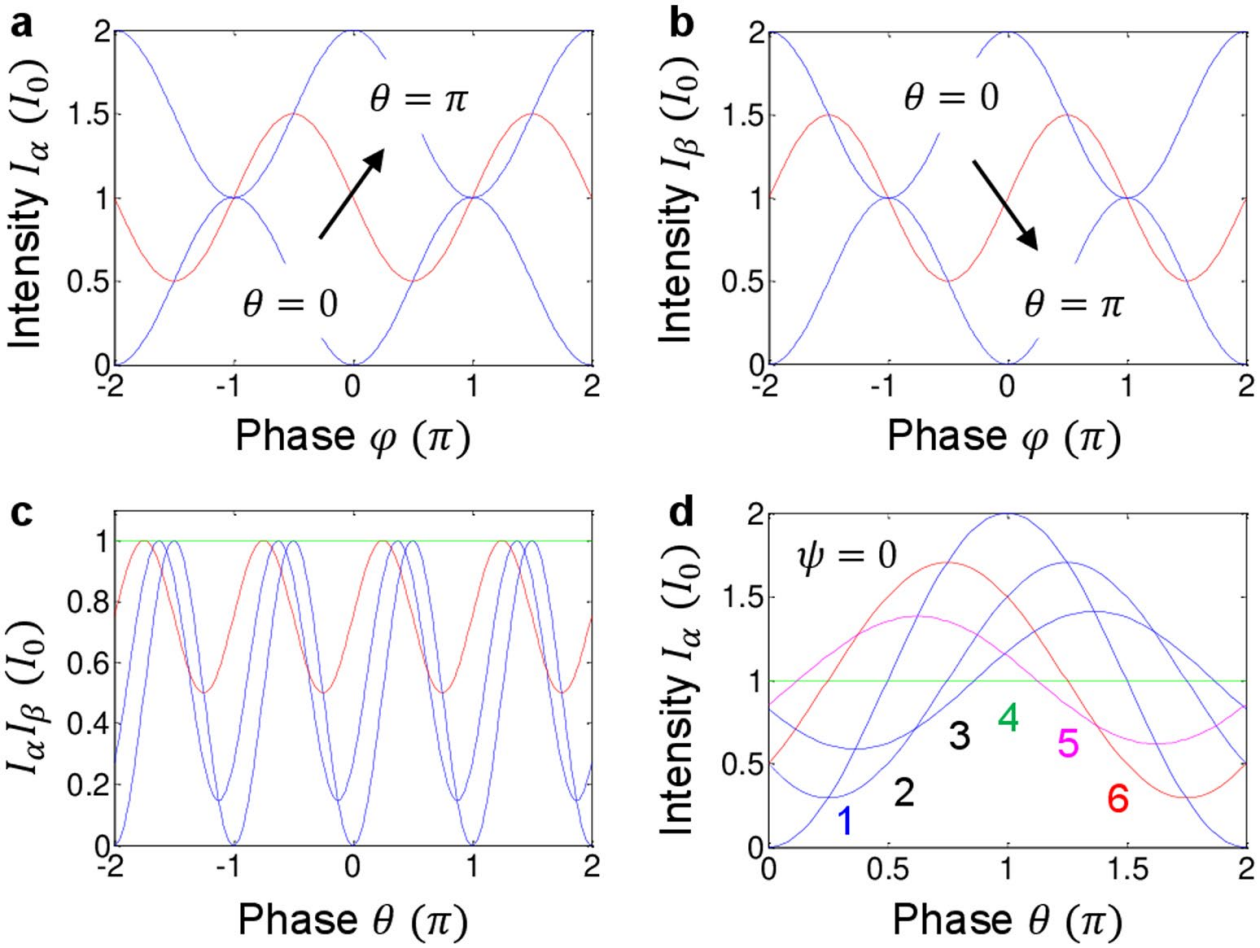
Numerical calculations for euations (3)–(5). (**a**) and (**b**) Intensities ${I}_{\alpha }$ and ${I}_{\beta }$ for $\uptheta =0$ (blule), $\uptheta =\uppi /2$ (red), $\uptheta =\uppi$ (dotted). (**c**) Intensity product ${I}_{\alpha }{I}_{\beta }$ versus phase $\uptheta$ for $\mathrm{\varphi }=0$ with $\uppsi =0$ (blue), $\uppsi =\uppi /4$ (dotted), $\uppsi =\uppi /2$ (red), and $\uppsi =\uppi$ (green). (**d**) ${I}_{\alpha }$ for $\mathrm{\varphi }=0$ (blue); $\mathrm{\varphi }=\uppi /2$ (dotted); $\mathrm{\varphi }=3\uppi /4$ (dashed); $\mathrm{\varphi }=\uppi$ (green); $\mathrm{\varphi }=5\uppi /4$ (magenta); $\mathrm{\varphi }=3\uppi /2$ (red). The numbers indicate increasing order of $\mathrm{\varphi }$.

Figure 3a,b represent output intensities as a function of $\mathrm{\varphi }$ for different $\uptheta$ for Eqs. (4) and (5). For these, Bob’s phase is fixed at $\uppsi =0$. As expected, the output intensities show constrasting results, but swings as $\uptheta$ varies. This swing effect is due to the H–H correlation indpenedent of the relative phase $\left|\varphi -\psi \right|$ as a common background. Although the global phase control by $\uptheta$ in one path (Bob) with resepct to another ($\mathrm{Alice}$) has no effect on the direct coinicidence measurements in Fig. 2a as shown in Eq. (3) ^1–16^, it affects the quantum features in an interferometric system of Fig. 1b (see also Fig. S1 of the Supplementary Informaton).

For this in Fig. 3c, both intensities ${I}_{\alpha }$ and ${I}_{\beta }$ in Fig. 3a,b are multiplied as a function of $\uptheta$ for different values of $\uppsi$ and fixed $\mathrm{\varphi }=0$ (see also Fig. S2 of the Supplementary Information). If the anticorrelation condition is satisfied as shown in the blue curve at $\uptheta =\pm \mathrm{n\pi }$ for $\mathrm{\varphi }=\uppsi =0$, photon bunching-caused anticorrelation occurs at every $\uptheta =\pm \mathrm{n\pi }$, resulting in a quantum feature^23^. The degraded anticorrelation such as in the red curve ($\uppsi =\frac{\uppi }{2};\mathrm{ \varphi }=0)$ can be fixed if $\mathrm{\varphi }$ is compensated to $\mathrm{\varphi }=\uppsi$ (see the bottom pannels of Fig. S2 of the Supplementary Information). However, there is no way to fix the correlation degradation if $\mathrm{\varphi }\ne\uppsi$ $(\mathrm{\varphi }\ne\uppsi \pm\uppi )$. As a result, the main parameters for Franson correlation are $\mathrm{\varphi }$ and $\uppsi$ even in an interferometric system. This is the fundamental physics of Franson-type nonlocal correaltion how it can be used for quantum key distribution^14–16^.

Figure 3d shows ${I}_{\alpha }$ versus $\uptheta$ for different $\mathrm{\varphi s}$ at fixed $\uppsi =0$, where phase $\mathrm{\varphi }$ increases from 0 (blue curve) toward $2\uppi$ as indicated by the increasing numbers (color matched). As analyzed in Fig. 3c, loss of anticorrelation or degradation of Franson correlation is due to violaiton of the anticorrelation condition: $\mathrm{\varphi }\ne\uppsi ;\mathrm{ \varphi }\ne\uppsi \pm\uppi$. This shows the nonlocal phase-correlation relation between $\uppsi$ (Bob) and $\mathrm{\varphi }$ (Alice). If $\uptheta (=\upeta +\upxi )$ is shifted by $\uppi /2$, then either Bob’s phase $\uppsi$ or Alice’s $\mathrm{\varphi }$ needs to be compensated by the same amount of shift for the condition of $\mathrm{\varphi }=\uppsi +\uptheta$ (see Fig. S3 of the Supplementary Information). However, the $\uptheta$ compensation cannot retrieve the visibility fully unless the anticorrelation condition between $\mathrm{\varphi }$ and $\uppsi$ is satisfied as shown in Fig. 3c. This means that the quantumness of nonlocal correlation is mainly related with the randomness-based superposition between two noninterfering PBS-BS MZIs via phase coherence. By the way, another set of PBS-BS MZI outputs A’ and B’ in Fig. 1 also results in the same features as Eqs. (3)–(6) (see Figs. S4 and S5 of the Supplementary Information): ${{I}_{{\alpha }^{^{\prime}}}=I}_{\alpha }$; ${{I}_{{\beta }^{^{\prime}}}=I}_{\beta }$. The related experimental demonstrations for Fig. 1b are shown separately^35^.

## Conclusion

In conclusion, the origin of entanglement or nonlocal correlation observed in Franson-type correlation was investigated for a coherence model using random polarization bases. To make the classical model similar to the original Franson correlation, random generation of polarized photons was provided by a macroscopic superposition technique using PBS-BS noninterfering MZI scheme, which is conceptually equivalent to the original Franson scheme based on path choices. From the analytical calculations, the first conclusion regarding the origin of Franson correlation was found in the random polarization basis. From numerical simulations, the second conclusion regarding the origin of Franson correlation was found in a definite phase relationship between polarization correlated photons directing to both parties. Lastly, the analytically obtained Franson correlation for the coherence model of the PBS-BS noninterfering MZIs was tested for anticorrelation, the so-called Hong-Ou-Mandel dip, where the two remotely separated but coherently coupled polarization-correlated output photons (or fields) were interfered on a BS. From this, the same phase relationship for anticorrelation was resulted in between two noninterfering MZIs. Like conventional understanding of nonlocal correlation based on random-basis superposition in SPDC-generated photon pairs, the same results were obtained in the present coherence model. Thus, the fundamental question for Franson-type nonlocal correlation was answered for correct definition of quantumness in terms of basis randomness rather than individual single photons. This answer paves a road to coherence quantum information for deterministic and macroscopic quantum technologies.

## Methods

The numerical calculations in Figs. 2 and 3 were performed by Matlab using equations appeared in the text. The data that support the findings of this study are available from the corresponding author upon reasonable request.

## Supplementary Information

Supplementary Information.

## Data Availability

The data presented in the figures of this Article are available from the corresponding author upon reasonable request.

## Code availability

All custom code used to support claims and analysis presented in this Article is available available from the corresponding author upon reasonable request.

## References

[1] Bell J (1964). On the Einstein Podolsky Rosen paradox. Physics.

[2] Clauser JF, Horne MA, Shimony A, Holt RA (1969). Proposed experiment to test local hidden-variable theories. Phys. Rev. Lett..

[3] Shih YH, Alley CO (1988). New type of Einstein-Podolsky-Rosen-Bohm experiment using pairs of light quanta produced by optical parametric down conversion. Phys. Rev. Lett..

[4] Brunner N (2014). Bell nonlocality. Rev. Mod. Phys..

[5] Hensen B (2015). Loophole-free Bell inequality violation using electron spins separated by 1.3 kilometers. Nature.

[6] Kwait PG, Mattle K, Weinfurter H, Zeilinger A (1995). New high-intensity source of polarization-entangled photon pairs. Phys. Rev. Lett..

[7] Bouwmeester D, Pan J-W, Mattle K, Eibl M, Weinfurter H, Zeilinger A (1997). Experimental quantum teleportation. Nature.

[8] Rowe MA, Kielpinski D, Meyer V, Sackett CA, Itano WM, Monroe C, Wineland DJ (2001). Experimental violation of a Bell’s inequality with efficient detection. Nature.

[9] Weihs G, Jennewein T, Simon C, Weinfurter H, Zeilinger A (1998). Violation of Bell’s inequality under strict Einstein locality conditions. Phys. Rev. Lett..

[10] Kim Y-H, Kulik SP, Shih Y (2001). Quantum teleportation of polarization state with a complete Bell state measurement. Phys. Rev. Lett..

[11] Tittel W, Brendel J, Zbinden H, Gisin N (2000). Quantum cryptography using entangled pohotons in energy-time Bell states. Phys. Rev. Lett..

[12] Kwiat PG, Steinberg AM, Chiao RY (1993). High-visibility interference in a Bell-inequality experiment for energy and time. Phys. Rev. A.

[13] Franson JD (1989). Bell inequality for position and time. Phys. Rev. Lett..

[14] Tapaster PR, Rarity JG, Owens PCM (1994). Violation of Bell’s inequality over 4 km of optical fiber. Phys. Rev. Lett..

[15] Brendel J, Gisin N, Tittel W, Zbinden H (1999). Pulsed energy-time entangled twin-photon source for quantum communication. Phys. Rev. Lett..

[16] Martin A, Kaiser F, Vernier A, Beveratos A, Scarani V, Tanzilli S (2013). Cross time-bin photonic entanglement for quantum key distribution. Phys. Rev. A.

[17] Tebyanian H, Zahidy M, Avesani M, Stanco A, Villoresi P, Vallone G (2021). Semi-device independent randomness generation based on quantum state’s indistinguishability. Quant. Sci. Tech..

[18] Han B-S, Yang Y, Bo W-J, Tang H (2020). Global dynamics of a Lotka-Volterra competition diffusion system with nonlocal effects. Int. J. Bifurc. Chaos.

[19] Chang M-X, Han B-S, Fan X-M (2021). Spatiotemporal dynamics for a Belousov–Zhabotinsky reaction-diffusion system with nonlocal effects. Appl. Analysis.

[20] Han B-S, Wang Z-C (2016). Traveling wave solutions in a nonlocal reaction-diffusion population model. Commun. Pure and Appl. Analysis.

[21] Han B-S, Chang M-X, Bo W-J (2022). Traveling waves for a Belousov–Zhabotinsky reaction-diffusion system with nonlocal effect. Nonlinear Anal. Real World Appl..

[22] Bohm D (1979). Quantum theory.

[23] Hong CK, Ou ZY, Mandel L (1987). Measurement of subpicosecond time intervals between two photons by interface. Phys. Rev. Lett..

[24] Ham BS (2020). The origin of anticorrelation for photon bunching on a beam splitter. Sci. Rep..

[25] Solano E, Matos Filho RL, Zagury N (1999). Deterministic Bell states and measurement of motional state of two trapped ions. Phys. Rev. A.

[26] Pfleegor RL, Mandel L (1967). Interference of independent photon beams. Phys. Rev..

[27] Deng Y-H (2019). Quantum interference between light sources separated by 150 million kilometers. Phys. Rev. Lett..

[28] Ham BS (2021). Macroscopically entangled light fields. Sci. Rep..

[29] Fleischhauer M, Lukin MD (2020). Dark-state polaritons in electromagnetically induced transparency. Phys. Rev. Lett..

[30] Monroe C, Meekhof DM, King BE, Wineland DJ (1996). Schrodinger cat” superposition sate of an atom. Science.

[31] Henry M (1981). Fresnel-Arago laws for interference in polarized light: A demonstration experiment. Am. J. Phys..

[32] Guo X, Mei Y, Du S (2017). Testing the Bell inequality on frequency-bin entangled photon pairs using time-resolved detection. Optica.

[33] Grangier P, Roger G, Aspect A (1986). Experimental evidence for a photon anticorrelation effect on a beam splitter: A new light on single-photon interference. Europhys. Lett..

[34] Xavier GB, von der Weid JP (2011). Stable single-photon interference in a 1 km fber-optic Mach-Zehnder interferometer with continuous phase adjustment. Opt. Lett..

[35] Kim, S. & Ham, B. S. Experimental demonstrations for randomness-based macroscopic Franson-type nonlocal correlation using coherently coupled photons. arXiv:2107.00302 (2021).

