# Supplementary material for: Randomness-based macroscopic Franson-type nonlocal correlation
Source: Sci Rep. 2022 Mar 8;12:3759. doi: 10.1038/s41598-022-07740-0 (PMC8904538; doi:10.1038/s41598-022-07740-0)
Supplement: Supplementary file 1 — Supplementary Information. [file 41598_2022_7740_MOESM1_ESM.pdf]

Supplementary Information for

Randomness-based macroscopic Franson-type nonlocal correlation by

BS Ham
GIST

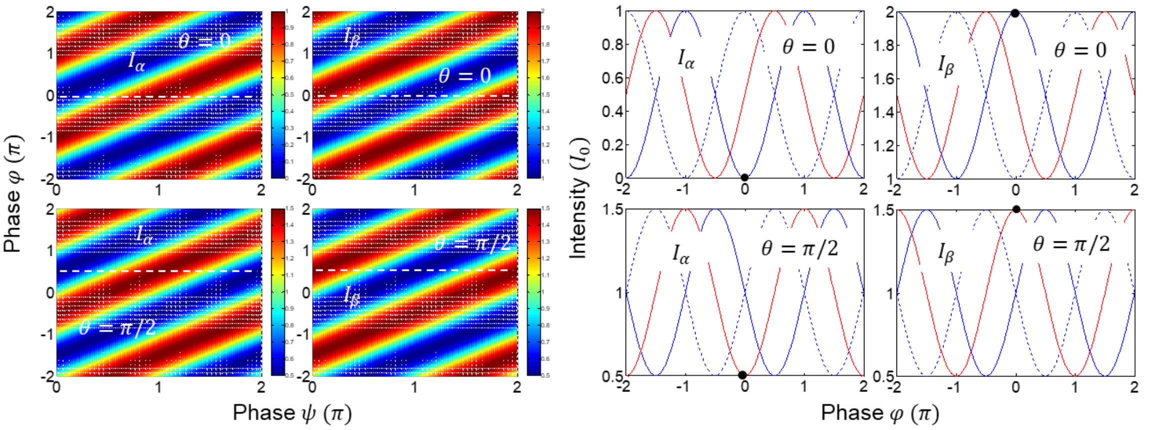

Fig. S1. Numerical simulations for the details of Figs. 2(e) and (f). Blue: $\psi = 0$, Red: $\psi = -\pi/2$, Dotted: $\psi = -\pi$.

The quantum feature defined in Bell inequality violation is typically represented by visibility greater than $1/\sqrt{2}$. In Fig. S1, the visibility of I_α is 100% but I_β is 50%, where this visibility swings across $\theta = 0$ as shown in Figs. 2(e) and (d). The main reason of this phenomenon is due to existence of common H-H correlation independent of the relative phase $|\varphi - \psi|$ as a common background. However, in terms of anticorrelation, this weird phenomenon disappears as shown in Fig. 2(g) for $\theta = 0$. If $\theta \neq 0$, then there is a loss of visibility violating anticorrelation condition of $\varphi = \psi + \theta$.

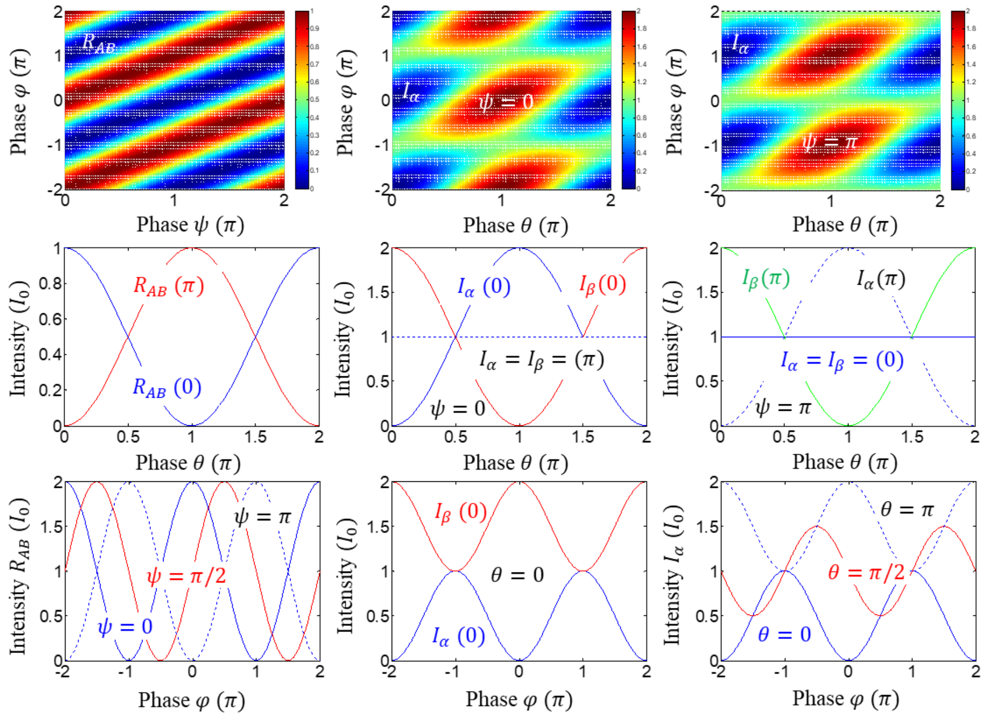

Fig. S2. Numerical simulations for the details of Fig. 2(e) and (f). The value in parenthesis of intensity in the middle (bottom) pannels is for φ (ψ).

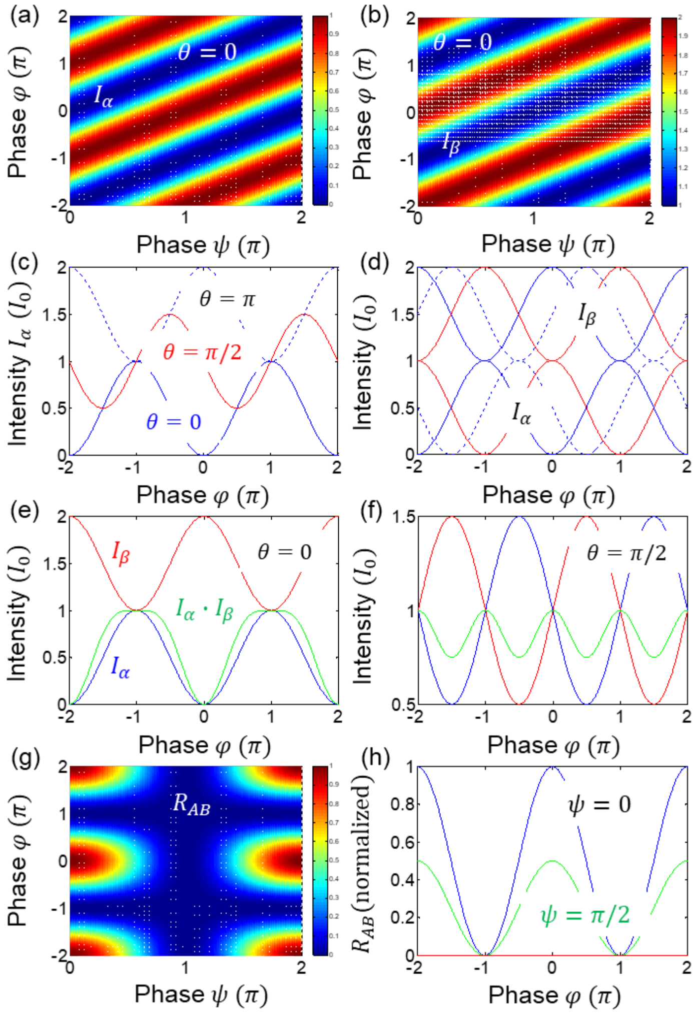

Fig. S3. Numerical simulations for the details of Fig. 2(h).

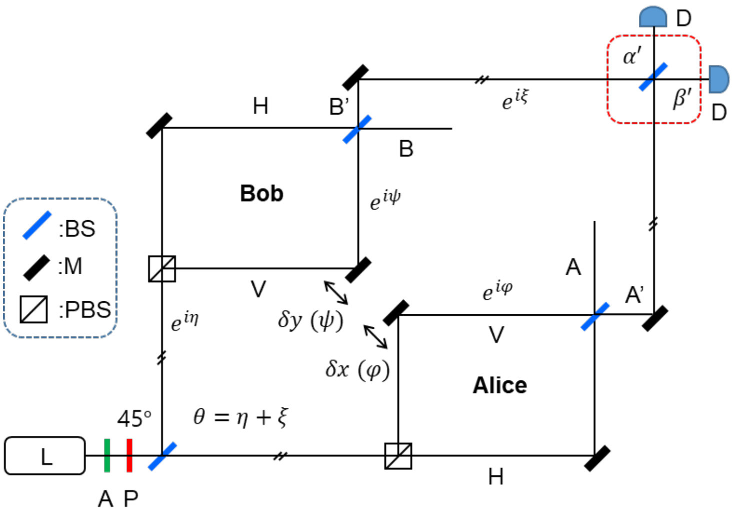

Fig. S4. Schematic of the coherence version of Franson-type nonlocal correlation. See Fig. 1 for details.

Analytic representations for Fig. S1 are as follows (see the main text for detailed information):

$$|\Psi\rangle_{A'} = (i|H\rangle_A + e^{i\varphi}|V\rangle_A)/\sqrt{2}, \tag{S1}$$

$$|\Psi\rangle_{B'} = ie^{i\theta}\left(i|H\rangle_B + e^{i\psi}|V\rangle_B\right)/\sqrt{2}, \tag{S2}$$

where amplitude of H and V is equivalent to E_0, and the term of $ie^{i\theta}$ is due to the overall relative path-length difference between Alice and Bob from the light source L. Due to the Fresnel-Arago law, H-V interference terms for both sides are automatically removed [35]. Thus, the interference between A' and B' on a BS results in the follows:

$$\begin{aligned} |\alpha'\rangle &= (|\Psi\rangle_{A'} + i|\Psi\rangle_{B'})/\sqrt{2} \\ &= [i|H\rangle_A + e^{i\varphi}|V\rangle_A] - \left[\left(i|H\rangle_B + e^{i\psi}|V\rangle_B\right)e^{i\theta}\right], \end{aligned} \tag{S3}$$

$$\begin{aligned} |\beta'\rangle &= (i|\Psi\rangle_{A'} + |\Psi\rangle_{B'})/\sqrt{2}, \\ &= [i|H\rangle_A + e^{i\varphi}|V\rangle_A] + \left[\left(i|H\rangle_B + e^{i\psi}|V\rangle_B\right)e^{i\theta}\right], \end{aligned} \tag{S4}$$

$$\begin{aligned} I_{\alpha'} &= \tfrac{1}{4}\left\{\left[\left(iH_A + e^{i\varphi}V_A - e^{i\theta}\left(iH_B + e^{i\psi}V_B\right)\right)\right]\left[\left(-iH_A^* + e^{-i\varphi}V_A^* - e^{-i\theta}\left(-iH_B^* + e^{-i\psi}V_B^*\right)\right)\right]\right\} \\ &= \tfrac{1}{4}\left[H_AH_A^* + V_AV_A^* + H_BH_B^* + V_BV_B^* - \left(H_AH_B^* + V_AV_B^*e^{i(\varphi-\psi)}\right)e^{-i\theta} - \left(H_BH_A^* + V_BV_A^*e^{-i(\varphi-\psi)}\right)e^{i\theta}\right] \\ &= \tfrac{I_0}{2}[2 - \cos(\theta) - \cos(\varphi - \psi - \theta)], \end{aligned} \tag{S5}$$

$$\begin{aligned} I_{\beta'} &= \tfrac{1}{4}\left\{\left[-\left(iH_A + e^{i\varphi}V_A - e^{i\theta}\left(iH_B + e^{i\psi}V_B\right)\right)\right]\left[-i\left(-iH_A^* + e^{-i\varphi}V_A^* - e^{-i\theta}\left(-iH_B^* + e^{-i\psi}V_B^*\right)\right)\right]\right\} \\ &= \tfrac{1}{4}\left[H_AH_A^* + V_AV_A^* + H_BH_B^* + V_BV_B^* - \left(H_AH_B^* + V_AV_B^*e^{i(\varphi-\psi)}\right)e^{-i\theta} - \left(H_BH_A^* + V_BV_A^*e^{-i(\varphi-\psi)}\right)e^{i\theta}\right] \\ &= \tfrac{I_0}{2}[2 + \cos(\theta) + \cos(\varphi - \psi - \theta)]. \end{aligned} \tag{S6}$$

Thus, $I_{\beta'} = I_\beta$ and $I_{\alpha'} = I_\alpha$ (see equations (4) and (5) I the main text). The coincidence detection rate is also $R_{A'B'} = R_{AB}$ (see equation (3) in the main text).

Figure S2 shows numerical simulations for equations (S5) and (S6), which is the same as Fig. 2. Figure S2(a) is for the overlapped view of both intensities of $I_{\alpha'}$ and $I_{\beta'}$ with respect to φ and ψ for θ = 0. Figure S2(b) is for Fig. S2(a) for θ = ψ = 0, satisfying a zero phase shift between Alice and Bob (see the big MZI square in

Fig. S1). Thus, the general characteristics of energy conservation in an MZI scheme of Fig. S1 are satisfied between Alice and Bob.

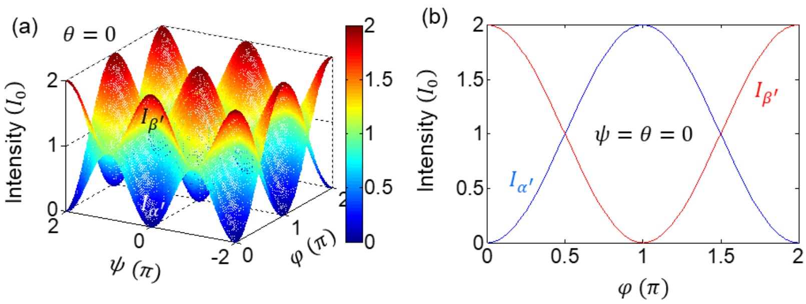

Fig. S5. Numerical simulations for equations (5) and (6).
